# HIV-1 Genetic Variation Resulting in the Development of New Quasispecies Continues to Be Encountered in the Peripheral Blood of Well-Suppressed Patients

**DOI:** 10.1371/journal.pone.0155382

**Published:** 2016-05-19

**Authors:** Will Dampier, Michael R. Nonnemacher, Joshua Mell, Joshua Earl, Garth D. Ehrlich, Vanessa Pirrone, Benjamas Aiamkitsumrit, Wen Zhong, Katherine Kercher, Shendra Passic, Jean W. Williams, Jeffrey M. Jacobson, Brian Wigdahl

**Affiliations:** 1 Department of Microbiology and Immunology, Drexel University College of Medicine, Philadelphia, Pennsylvania, United States of America; 2 Center for Molecular Virology and Translational Neuroscience, Institute for Molecular Medicine and Infectious Disease, Drexel University College of Medicine, Philadelphia, Pennsylvania, United States of America; 3 School of Biomedical Engineering, Science, and Health Systems, Drexel University, Philadelphia, Pennsylvania, United States of America; 4 Center for Genomic Sciences, Institute for Molecular Medicine and Infectious Disease, Drexel University College of Medicine, Philadelphia, Pennsylvania, United States of America; 5 Center for Advanced Microbial Processing, Institute for Molecular Medicine and Infectious Disease, Drexel University College of Medicine, Philadelphia, Pennsylvania, United States of America; 6 Department of Medicine, Division of Infectious Diseases and HIV Medicine, Drexel University College of Medicine, Philadelphia, Pennsylvania, United States of America; 7 Center for Clinical and Translational Medicine, Institute for Molecular Medicine and Infectious Disease, Drexel University College of Medicine, Philadelphia, Pennsylvania, United States of America; 8 Sidney Kimmel Cancer Center, Thomas Jefferson University, Philadelphia, PA, United States of America; George Mason University, UNITED STATES

## Abstract

As a result of antiretroviral therapeutic strategies, human immunodeficiency virus type 1 (HIV-1) infection has become a long-term clinically manageable chronic disease for many infected individuals. However, despite this progress in therapeutic control, including undetectable viral loads and CD4^+^ T-cell counts in the normal range, viral mutations continue to accumulate in the peripheral blood compartment over time, indicating either low level reactivation and/or replication. Using patients from the Drexel Medicine CNS AIDS Research and Eradication Study (CARES) Cohort, whom have been sampled longitudinally for more than 7 years, genetic change was modeled against to the dominant integrated proviral quasispecies with respect to selection pressures such as therapeutic interventions, AIDS defining illnesses, and other factors. Phylogenetic methods based on the sequences of the LTR and *tat* exon 1 of the HIV-1 proviral DNA quasispecies were used to obtain an estimate of an average mutation rate of 5.3 nucleotides (nt)/kilobasepair (kb)/year (yr) prior to initiation of antiretroviral therapy (ART). Following ART the baseline mutation rate was reduced to an average of 1.02 nt/kb/yr. The post-ART baseline rate of genetic change, however, appears to be unique for each patient. These studies represent our initial steps in quantifying rates of genetic change among HIV-1 quasispecies using longitudinally sampled sequences from patients at different stages of disease both before and after initiation of combination ART. Notably, while long-term ART reduced the estimated mutation rates in the vast majority of patients studied, there was still measurable HIV-1 mutation even in patients with no detectable virus by standard quantitative assays. Determining the factors that affect HIV-1 mutation rates in the peripheral blood may lead to elucidation of the mechanisms associated with changes in HIV-1 disease severity.

## Introduction

The introduction of combination therapeutics have extended the life expectancy of those infected with the human immunodeficiency virus type 1 (HIV-1) for many years past what would have been observed even a decade ago. Due to the mutable nature of the HIV-1 genome, longer life expectancy and associated selective pressures has led to increased opportunities for the emergence of viral genetic variants that escape therapy [1, 2], contribute to neurocognitive decline [3–5], or result in more or less frequent activation/reactivation of persistent/latent infection [6], among many other potential complications resulting from a large number of well-known comorbidities [7]. As such, it is important to understand the nature of viral genetic variation within individual hosts both prior to and after the initiation of combination antiretroviral therapy (cART) and perhaps just as importantly, among the various combination therapeutic regimens.

Over the past several years, a great deal of attention has focused on the fate of the infecting viral inoculum and the cells that are initially targeted in the genital tract, regional lymph nodes, and peripheral blood during the early acute stages of infection. The general conclusions from studies of heterosexual transmission have indicated that infection is typically acquired from a single viral genotype (80% of cases), and the transmitted virion typically utilizes the CCR5 coreceptor (designated an R5 virus), which replicates well in CD4+ T-cell cultures but not monocyte-derived macrophage cultures [8–11]. Although intra-host genetic bottlenecks have been identified by a number of mechanisms and at different anatomic sites following the initial encounter, these results are consistent with a small founder population and host tropisms that have previously been observed in the simian immunodeficiency virus (SIV) rhesus macaque model [12, 13].

Viral genetic variation over time in a patient is likely the result of at least three processes. The first process involves the introduction of novel mutations between the entry and integration steps of the viral life cycle, due to errors in the proof-reading mechanism of HIV-1 reverse transcriptase [14]. These errors have been estimated to occur at an average rate of 0.1 mutations per genome per cell generation as measured by single-round integration experiments [14]. Proofreading errors results in an average mutation rate of ~2 nucleotides per kilobase per year per host, as measured in a large-scale phylogenetic analysis of the Los Alamos database [15]. The high mutation rates of reverse transcription indicate that 1 in 10 cellular infection events results in a novel, replication competent, viral genome being integrated into the host cell. This results in “clouds” of similar genomes that collectively span a large portion of the replication competent sequence space [16], each of which is referred to as a “quasispecies”. Through natural selection, viral genomes harboring beneficial variants will increase in frequency. However, individual patients present a complex and variable fitness landscape, and thus HIV’s high mutation rate allows it to maintain a high degree of diversity to evade immune pressure, antiretroviral therapy, and other host-specific selective pressures [17].

The second major modulator of viral genetic variation within a host centers on how variant sequences are affected by selective pressures resulting from immune responses, therapeutic interventions, drug abuse, and other environmental factors, which all affect the frequency of preexisting viral variants. In most cases, this leads to a predominant sequence comprising between 20% to 80% of the total number of viral quasispecies sequences along with potentially dozens of lower frequency variant sequences [18]. Viral quasispecies that contain new mutations or preexisting low frequency viral quasispecies that acquire drug resistance alleles or immune escape variants can increase in frequency to predominate when the selective pressures change over time [1, 19, 20]. Due to changes in patient lifestyles (e.g. lapses in therapeutic compliance, changing substance abuse patterns, or general health), selective pressure can also change, shifting the frequency of individual viral genetic variants within individual HIV-1-infected patients.

The third general source of viral genetic variation in the peripheral blood is based on the periodic escape or release of HIV-1 quasispecies from cellular and anatomical reservoirs harboring virus. Subsequent or continued replication of such previously compartmentalized viruses after transition to the peripheral blood allows for the seemingly hidden evolution of new quasispecies in the peripheral blood. During the acute infectious process when the virus achieves very high viral loads it seeds several anatomic reservoirs including the brain [21], lung [22], gut, as well as other locations including the bone marrow [23]. Integration into the host cell’s chromosome of the HIV-1 genome as a provirus allows it to remain latent in resting memory CD4+ T cells where it can evade most selective pressures of the immune system [24, 25]. New viral quasispecies and their cognate viral particles can emerge from these reservoirs through either activation of the latent memory CD4+ T-cell population, or from resident cell populations within the anatomic reservoirs through unknown mechanisms. Understanding the clinical factors that influence activation and viral release from more therapeutically refractile reservoirs will be crucial to the success of the next generation of therapeutics such as shock-and-kill and related strategies [26], therapeutic vaccine development [27], and proviral excision techniques that hold great promise to eradicate HIV infection [28].

The Drexel Medicine CNS AIDS Research and Eradication Study (CARES) Cohort in Philadelphia, PA is a unique resource to examine the interplay between these three biologic processes that impact the course of viral genetic variation in individual infected patients. More that 550 HIV-1-infected patients within the CARES Cohort have been longitudinally sampled with return visits scheduled approximately every six months. PCR-based Sanger sequencing has been utilized to sample the viral sequence from multiple genomic regions of integrated HIV-1 provirus populations, and this has provided for a detailed inspection of the sequential variation of the dominant proviral quasispecies across many HIV-1-infected patients [29–32].

Modern computational methods are able to integrate multiple time-stamped sequences into a phylogenetic reconstruction of current and past divergence histories. Bayesian Estimation of Ancestors by Sampling Trees (BEAST) is a well-established tool used to estimate the divergence time of historic sequences, as well as lineage-specific evolutionary rates using a relaxed molecular clock assumption [33]. It has also been used to estimate HIV-1 transmission networks in Kenya [34] and the emergence of novel HIV-1 variants in China [35]. Josefsson and coworkers examined the HIV-1 mutation rates in the resting memory CD4+ T-cell and gut reservoirs of several patients that were well-suppressed on long-term ART and found a rate of <3 mutations per gene over a 4–12 year time-period, depending on the gene sampled and time between measurements [36].

In this study, the LTR and *tat* exon 1 regions of 36 patients in the CARES Cohort were sampled over a six-year period and BEAST phylogenetic analyses were performed to determine how the mutation rates changed over time, and how clinical factors may affect mutation rates.

## Materials and Methods

Patients enrolled in the Drexel Medicine CARES Cohort were recruited from the Partnership Comprehensive Care Practice of the Division of Infectious Disease and HIV Medicine in the Department of Medicine at Drexel University College of Medicine (Philadelphia, Pennsylvania, USA) and the Center for Clinical and Translational Medicine in the Drexel Institute for Molecular Medicine and Infectious Disease. Patients from this Cohort encompass a broad range of clinical severities; however, in general, the CARES Cohort consists primarily of African American males that have been infected for a median of 15.9 [15.6–16.7] years and who have been on therapy for a median of 13.5 [12.9–14.3] years (Table 1). For the archival analysis presented herein, 36 patients were selected from the CARES Cohort using the following inclusion criteria with each patient having: (1) eight or more visits, (2) at least one visit naïve to therapy, and (3) at least 2 years on ART. Patients were excluded if they switched ART therapy while in the Cohort or if they have ever admitted to IV drug use (rationale discussed below).

**Table 1:** Demographics of the entire CARES Cohort as well as the subset of patients that have been included in the CARES longitudinal arm and those that were used for confirmation by subcloning and sequencing. The values indicate the median and 95% confidence intervals. *Due to the longitudinal and archival nature of this dataset, the limit of detection with respect to viral load has changed but has ranged from <100 to <20.

Category	Note	Entire Cohort	Clone Sampling	Longitudinal Sampling
**Patient number**		506	31	36
**Age**		49 [47.8–49.1]	44 ± [41.8–46.0]	51.5 [49.8–54.2]
**Gender**	Male	62%	62%	59%
	Female	37%	37%	41%
**CD4 (cells/mL)**	Latest	522 [536–585]	494 [447–592]	675 [645–830]
	Nadir	223 [249–281]	199 [179–249]	212 [179–267]
**Viral Load (copies/uL)**	Latest	48 [48–18,200]	235 [48–442,000]	Undetectable*
	Peak	45,500 [151,000–313,000]	90,860 [122,000–786,000]	75,012 [84,700–221,000]
**Years Seropositive**		15.9 [15.6–16.7]	10.9 [10.5–14.2]	19.1 [18.2–21.8]
**Years on ART**		14.1 [12.9–14.3]	5.6 [3.22–724]	14.7 [12.5–16.8]

Whole blood was collected from each of the 36 patients, processed, and assessed as previously described [29]. This procedure was performed at the initial visit and at each subsequent return visit. Peripheral blood samples were used for drug screening, plasma analysis, and PBMC isolation as previously described [32].

The LTR and *tat* exon 1 regions were amplified from genomic DNA isolated from PBMCs with three different methods: (1) The “PCR Read” method which consisted of direct Sanger sequencing of a PCR product generated from genomic DNA; approximately 90% of the patient samples have a PCR R*ead*. (2) The “Clone Read” consisted of subcloning to isolate individual fragments from PCR products with subsequent sequencing of the clones; 31 patients had at least 10 clones and an average of 16 clones per sample (Table 1, column 2). (3) The “4.4 kb Fragment Read” consisted of amplifying a 4.4 kb fragment from the 3’-end of the viral genome followed by isolation of individual fragments by subcloning and Sanger sequencing; approximately 36% of patients have a “4.4 kb Fragment Read”. Greater detail on the PCR procedure has been described [29, 32]. The approximately 2,300 individual sequences used in this analysis were deposited in Genbank under Bioproject ID PRJNA318511.

### Phylogenetic reconstruction

Phylogenetic reconstruction of the evolutionary history of the HIV-1 sequences derived from the peripheral blood of each patient was performed using version 1.8.1 of the BEAST package [33]. This method uses a Bayesian Markov-Chain Monte-Carlo estimation technique to find the most likely tree given a set of sequences along with the provision of sampling dates and assumptions about the population size, root height, nucleotide distribution, as well other parameters as defined below. The assumptions behind the BEAST model are critical to fitting reasonable estimates to the data. The model was fit with a Bayesian skyline assumption [37] in which the effective population size was allowed to vary over 10 equally spaced points between the root and tip of the tree. The effective sample size was estimated as an exponentially distributed variable with a mean calculated from the viral loads of each patient. The root height was estimated from medical records of HIV infection and modeled as a truncated normally distributed variable with a standard deviation of 5 years and limited to their age on the left end and their first HIV-related medical event on the right. This assumption implies a sexual transmission route, and as such, patients who admitted to IV drug use or who had likely been infected by transfusion based on clinical history were excluded from the analysis because of a higher probability of having experienced a blood stream transmission event rather than transmission by sexual intercourse. The average mutation rate was modeled as a normally distributed variable, truncated at 0, with a mean of 2 mutations/kb/year with a standard deviation of +/- 1, based on previous experimental estimates [15]. Nucleotide substitution models were selected independently for each alignment using the Bayesian information selection criteria as implemented in the jModelTest 2 package [38]. All other parameters were left in the default mode.

Sequences were first aligned with the MUSCLE alignment tool using default parameters [39]. Simulations were run for 1,000,000 iterations with samplings every 1,000 chains. The maximum likelihood phylogenetic trees were reconstructed using the TreeAnnotator tool packaged with BEAST to estimate the tree topology and 95% confidence intervals on divergence times and mutation rates. The multiple alignments were also processed using the RAxML tool version 8.1.17 for bootstrapping [40]. Only splits that occurred in 80% of the trees were maintained. The maximum likelihood tree was compared to the tree generated by BEAST and those with less than 90% agreement in the complete phylogenetic topology were excluded from further analysis.

### Statistical Analysis

Trees were processed with the Dendropy [41] python package to measure mutation rate estimates for each branch. The mutation rates across all branches at a particular time point were aggregated by averaging the estimates across each branch that existed at that time point taking into account the confidence intervals provided by the BEAST analysis. Due to the longitudinal nature of this study, accounting for non-independence between measurements was non-trivial. Mixed effects models were used to account for the fixed effects of the measured variables in the presence of an infinite array of patient-specific random effects. All statistical analyses were performed with the MixedML package of the Statsmodels python toolset [http://statsmodels.sourceforge.net/].

## Results

### Use of PCR to directly amplify members of the HIV-1 quasispecies from genomic DNA

**Fig 1:**
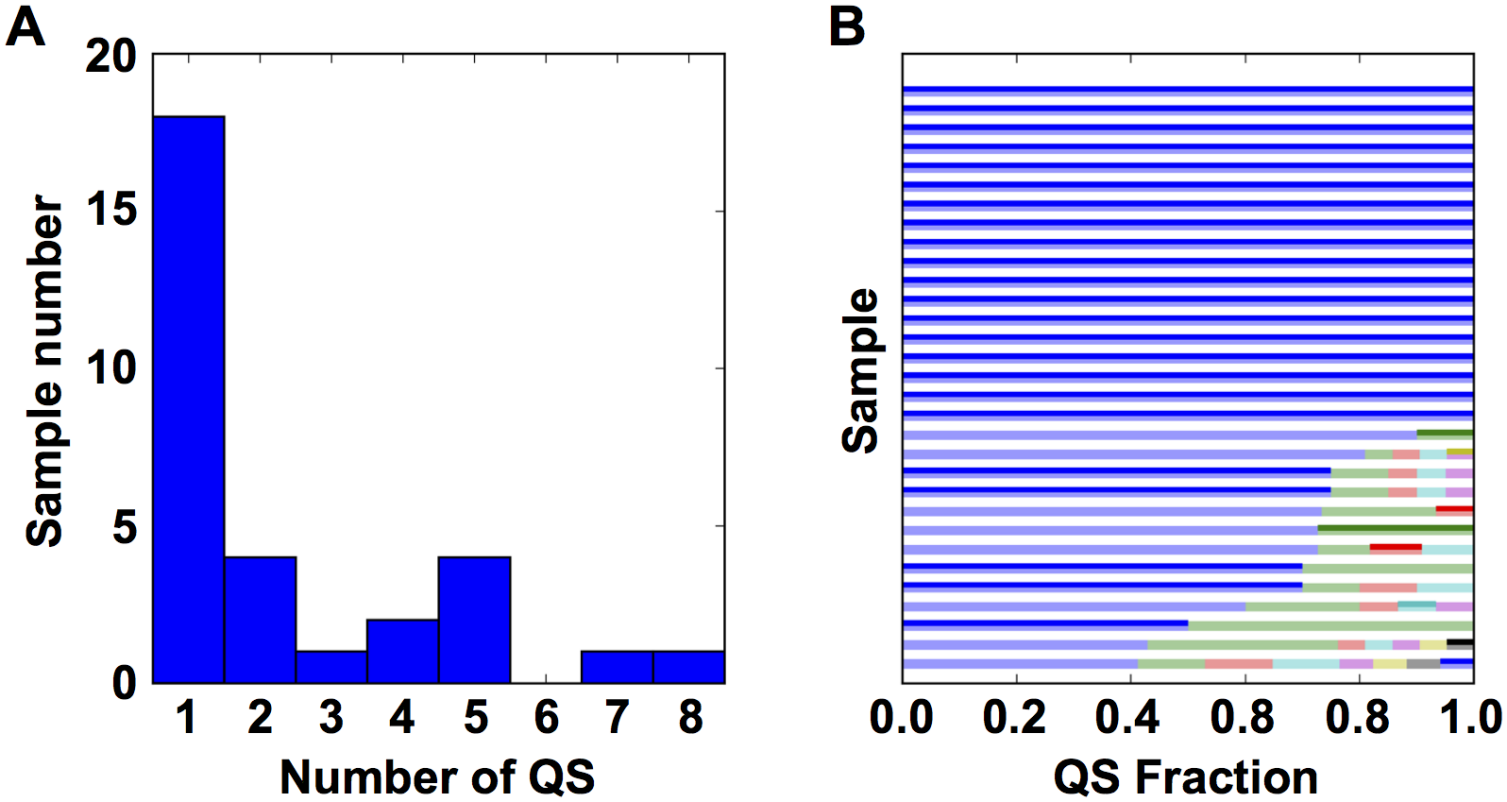
Direct sequencing of PCR products generated directly from genomic DNA very often returns the predominant genotype. **(A)** (Left) A histogram of the number of QS observed in each of the 31 samples with at least 10 Clone Reads. **(B)** A bar plot showing the fraction of the total observed QS for each unique Clone Read. Each horizontal bar represents one of the 31 samples with the width of the bar indicating the QS fraction. Each color in the bar indicates unique QS. Darker bars denote the QS that match the PCR Read.

In an effort to simplify the process of longitudinal sampling of HIV-1 quasispecies in the Drexel Medicine CARES Cohort, PCR amplification of specific regions of the HIV-1 genome was performed on genomic DNA and the resulting PCR products were directly sequenced without the traditional step of sub-cloning to isolate individual sequence fragments prior to sequencing the cloned product. To validate this direct PCR-based sequencing protocol, it was compared to the longer and more expensive sequencing strategy, in which PCR products were cloned, and several individual clones were sequenced. Towards this end, a set of 31 patient samples were subjected to the traditional sub-cloning PCR strategy [29, 32]. From these 31 samples, 507 total clones were obtained with an average of 16.3 Clone Reads per sample. By counting the number of unique sequences it was estimated that there were 2.32 QS per sample (Fig 1A) each representing 4% to 100% (mean 43%) of the observed QS (Fig 1B).

To further determine whether the PCR read generated a single member of the quasispecies or an amalgamation of the entire quasispecies, the PCR Read was compared to all Clone Reads. In all 31 samples (each with at least 10 Clone Reads) the PCR Read exactly matched at least one of the Clone Reads. Also of particular interest is whether the PCR Read matched the most predominant unique Clone Read. In 23/31 cases the PCR Read matched the predominant Clone Read (Fig 1B). While the PCR Read does not always provide a measurement of the Predominant Clone Read, it does provide a member of the quasispecies; as such it is possible to use these sequences for subsequent phylogenetic reconstructions.

### Longitudinal analysis of mutation rate

Using a subset of patients from the CARES Cohort, phylogenetic reconstruction using the BEAST analysis tool was performed to estimate the change in evolutionary rate over time. In brief, this process involved fitting a date-tipped maximum likelihood tree, in which the date at the root of the tree was fit to a probability model using the patient-reported infection date as a prior, A relaxed molecular clock assumption allowed for a variable mutation rate across each branch. Trees were generated for the LTR and *tat* exon 1. Due to the current sequencing progress in the Drexel Medicine CARES Cohort and the exclusion criteria discussed in Materials and Methods, this resulted in the construction of 84 trees for the LTR, 12 trees for *tat* exon 1, and 6 trees for *tat* exon 2. BEAST trees that disagreed with standard phylogenetic trees generated using RAxML were then excluded. This resulted in a high confidence set of 36 LTR trees and 8 *tat* exon 1 trees (Table 1, column 3).

**Fig 2:**
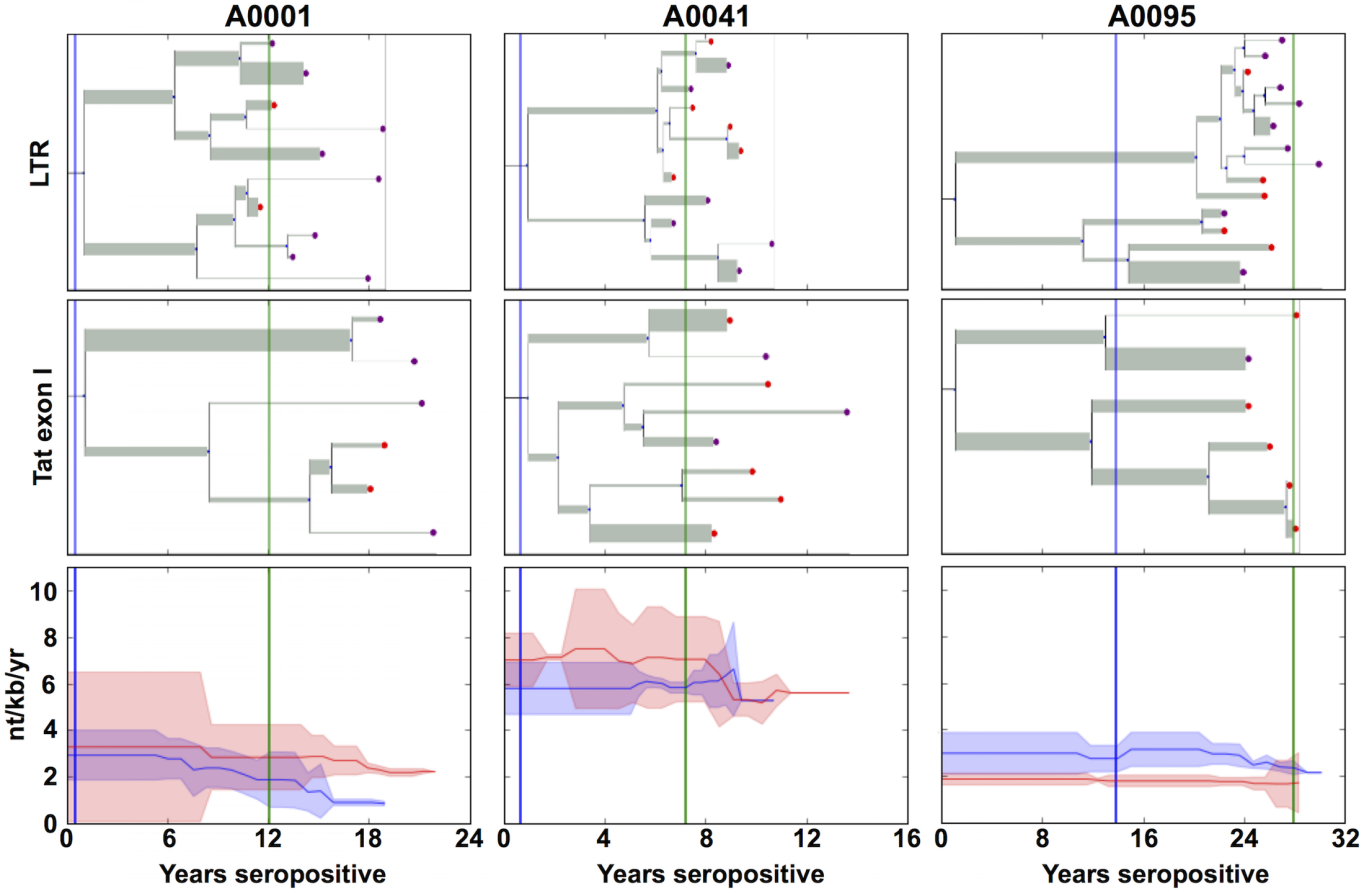
The mutation rate of HIV varies over time as determined by BEAST analysis. BEAST trees from three representative patients (A0001, A0041 and A0095) are shown for both the LTR **(Top)** and *tat* exon 1 **(Middle)** regions The width of the branch indicates the rate of mutation across the branch and the branch-length represents the time since divergence. The purple nodes indicate PCR Reads, the red nodes 4.4 Kb Fragment Reads, and the green nodes indicate Clone Reads. **(Bottom)** The time-series of each patient was synchronized such that the estimated time of infection for all samples have been shifted to 0 years. The red line shows the average mutation rate of *tat* exon 1 and the red shadow shows the 95% confidence interval of the estimate. The blue line shows the average mutation rate of the LTR and the blue shadow shows the 95% confidence interval of the estimate.

In order to estimate the time-varying rate of HIV-1 mutation in these patients, a summary method was developed to obtain a mutation rate. Using the date-tipped BEAST trees, the mutation rate was averaged across all existing branches at a particular time-point, taking into account the Bayesian credible intervals provided by the BEAST algorithm. The BEAST-constructed trees for the LTR and *tat* exon 1 regions of three representative patients are shown (Fig 2). Aggregated estimates of the mutation rate over time are shown for the same three patients (Fig 2).

The BEAST trees shown in Fig 2 are representative of all the patients and illustrate several known features of HIV-1 clinical disease progression. These particular three patients were chosen as those with the most sequences of both LTR and *tat* exon 1. All six trees show a major split within the first few years of infection. The *tat* exon 1 tree of patient A0095 shows sequential mutation in the predominant variant as evidenced by the branching pattern of the bottom arm of the tree. Importantly, in all six trees there is a marked decrease in the mutation rate once ART therapy has lowered viral loads to undetectable levels (green line), as indicated by narrower branches and a lower aggregated mutation rate.

Interestingly, Fig 2 also shows instances of sequences emerging from long-armed phylogenetic branches. These imply that either a variant has emerged from an unsampled reservoir, the reactivation of previously latent genomes, or a member of the quasispecies moving from low frequency to a higher frequency. The last two could be resolved by an increase in the number of samples with multiple reads while the first requires sampling all potential reservoirs. The complications surrounding these topics are reviewed in greater detail in the discussion.

**Fig 3:**
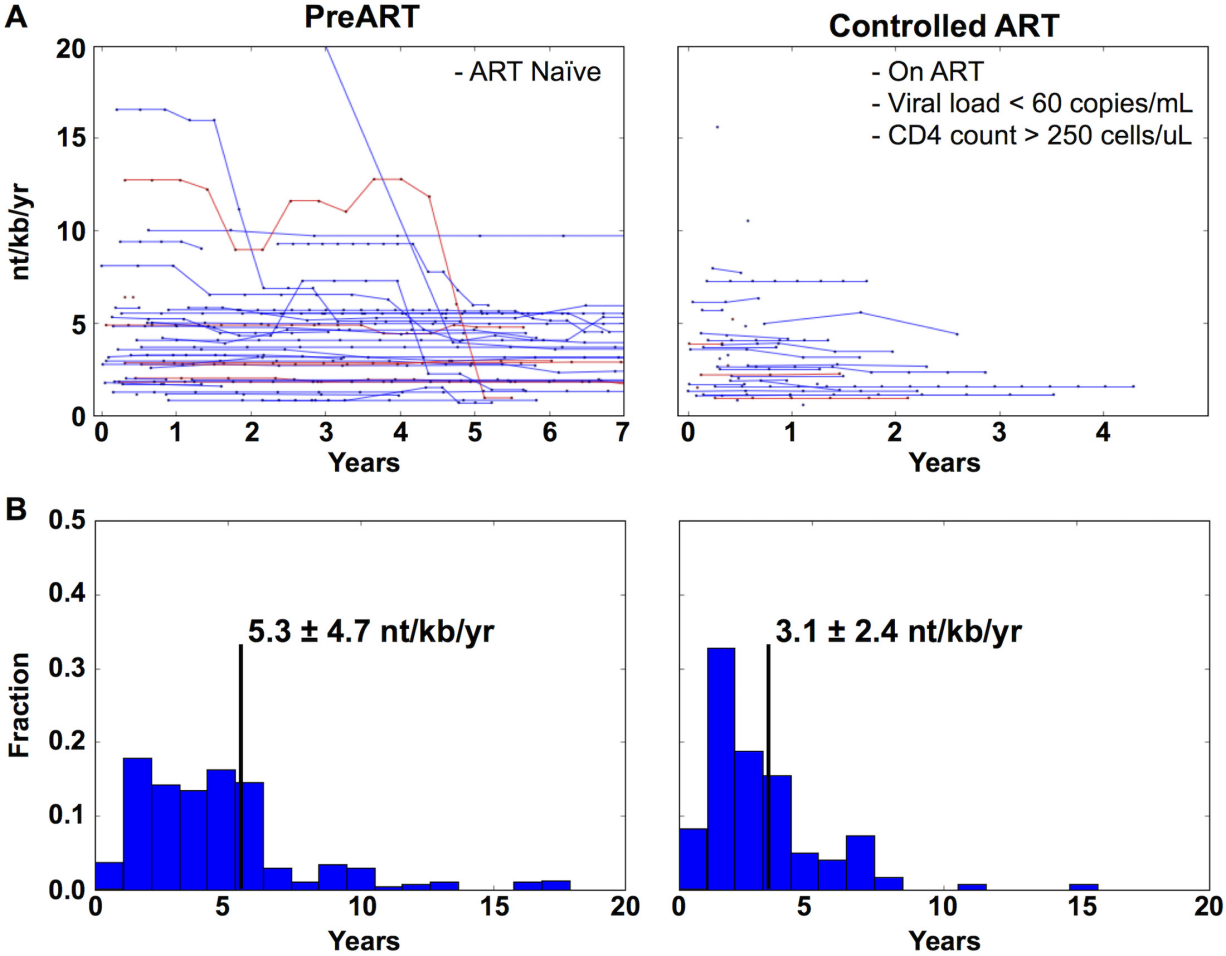
The mutation rate of HIV-1 is lowered by the introduction of ART but not to undetectable levels. **(A)** The time series of mutation rates for all patients (N = 36) that fall into the PreART Group and Suppressed ART Group. Red lines represent the mutation rate of *tat* exon I, while blue lines represent the LTR mutation rate. In order to synchronize each patient, their time points were aligned such that all patients in the PreART Group had their t = 0 set to their estimated time of infection. Patients in the Suppressed ART Group were synchronized such that t = 0 was set to the first clinical visit in which their viral loads dropped to undetectable levels and continued until either they switched therapy, lost viral control, or the current time was reached. **(B)** A normalized histogram of the mean mutation rate for each patient in either the PreART or Suppressed ART Group categories is shown. The black vertical line denotes the average rate.

To quantify the effect of ART therapy on the HIV-1 mutation rate, 36 patients were further analyzed. This subset of patients was selected such that more than one sample had been collected from the patient prior to the initiation of combination ART (PreART) and at least one sample had been collected, in which they had been on combination ART with undetectable viral loads. The samples from each patient were subdivided into the two groups. Specifically; the PreART Group refers to samples collected before the introduction of ART. The Suppressed ART Group refers to samples collected after the virus has been reduced to undetectable levels until therapy was changed or the latest sampled time point. As expected, the 36 patients have spent different lengths of time on combination ART and have been well-suppressed for different lengths of time. In order to synchronize the time series across multiple patients, the time points were synchronized using clinical markers. The PreART Group time points were measured from the reported HIV infection date (Fig 3A) while the Suppressed ART Group time points were measured from the first well-suppressed viral load measurement. In order to estimate the effect of ART, the mean mutation rate for each patient was calculated for the PreART Group and Suppressed ART Group and plotted as normalized histograms (Fig 3B). The mean mutation rates were calculated as 5.3 +/- 4.7, and 3.1 +/- 2.4 nt/kb/year for PreART and controlled ART, respectively. This suggested that, while viral control by combination ART is correlated with a decrease in mutation rate, the magnitude of this decrease was small.

To directly test the hypothesis that combination ART has a significant effect on the mutation rate, a mixed effects statistical model was built such that the mutation rate was due to three fixed effects: years seropositive, ART status, and genetic region sampled (LTR or *tat* exon 1). The random effects were assumed to be an independent intercept unique to each patient. This assumes that the virus in each patient has a unique basal mutation rate that is an aggregate of all unmeasured variables affecting viral mutation in that patient, thus accounting for repeated measure issues inherent with longitudinal data [42]. This model was compared to a null model, in which the only fixed effect was years seropositive. When compared to the null model, the alternative model has a likelihood ratio of 16.2 with an extra 2 degrees of freedom; which results in a chi-squared p-value = 7.38E-5. The estimated effect sizes of the parameters in this model are shown in Table 2. Based on this model, HIV-1 has a basal mutation rate of 5.21 mutations/kb/year and combination ART decreases this by 0.97 mutations/kb/year.

**Table 2:** The calculated effect sizes of fixed effects for the patients shown in Fig 3.

Parameter	nt/kb/yr	95% CI	p-value
Baseline (Intercept)	5.21	[4.17, 6.25]	9.68E-23
Patient Baseline (random effect)	0.0012	[-0.85, 0.85]	N/A
*Tat* exon 1 versus LTR	0.636	[0.067, 1.20]	2.85E-02
Suppressed ART	-0.972	[-1.35, -0.592]	5.47E-07
Years Seropositive	-0.064	[-0.088, -0.041]	6.31E-08

Numerous other clinical parameters contained within the Drexel Medicine CARES Cohort database were examined with respect to their correlation with time-varying mutation rate but no associations with a statistically significant effect were identified. Peak viral load and nadir CD4^+^ T-cell count showed no improvement in model fit (p>0.1). Age showed a mild correlation with mutation rate (p = 0.074); however, this may be due to its covariance with Years Seropositive; more data is needed to resolve this difference. Current viral loads (those within the detectable range), CD4^+^ T-cell, and CD8^+^ T-cell counts also did not correlate with the mutation rate (p>0.1).

The results, taken as a whole, indicate that the mutation rate was lowered by ART therapy in this dataset but that the overall effect is small and mutations remain ongoing even in the absence of detectable viral load for prolonged periods of time. Using this population of 36 patients sampled longitudinally for six years it is estimated that the effect of therapy on the mutation rate of HIV represents a 20% decrease. Notably, despite undetectable levels of viral loads the mutation rate has never been estimated to be zero. These results show that, even in the absence of detectable viral load, new genetic variation continues to arise in the proviral DNA population. Importantly, the analysis performed treated distinct lineages, or quasispecies, of provirus individually since lineage-specific mutation rates were estimated, therefore reactivation of latent reservoirs and patients infected by multiple distinct genotypes are not confounding the results.

## Discussion and Conclusions

We have hypothesized that the genetic variation over time within the integrated HIV-1 proviral genome in the infected cells derived from the peripheral blood represents (1) a combination of sequential mutations within quasispecies, (2) changes in the relative frequencies of distinct quasispecies, and (3) the reemergence of compartmentalized genomes leaking from viral reservoirs including the brain, gastrointestinal tract, and perhaps other tissues that may include the bone marrow. Longitudinal analysis of HIV-1 proviral sequences derived from patients in the Drexel Medicine CARES Cohort is beginning to quantify the relative contribution of each of these forces. In the present analysis, we have demonstrated that it is possible to construct date-tipped phylogenies and estimate changes in mutation rates in response to clinical factors/events.

Of the three potential sources of variation, this study focused on lineage-specific mutation rates and how these changed over time to ART. The sampling of Clone Reads performed on the 31 samples was insufficient to capture low frequency variants, usually defined as those below 1% [1, 20]. This makes it difficult to distinguish between the resurgence of a low-frequency variant and the reemergence of a historical compartmentalized differentially dormant or latent provirus. Future research will focus on disentangling the effect of all three modes of variation during the course of HIV-1 disease. This will be accomplished through the examination of similar longitudinal samples but utilizing next generation sequencing platforms including the Illumina NextSeq and Pacific Bioscience (PacBio) platforms to explore low frequency variants and their change in relative abundance over time.

Examining the release of virus from a “leaky” viral reservoir will require additional experimental approaches in addition to a greater depth of sequence coverage. For example, fluorescence-activated cell sorting can be used to separate cells to specifically examine the CD4+ memory T-cell compartment, a likely cellular reservoir [24, 25]. Examining a number of anatomical reservoirs using cerebrospinal fluid [21], GI tract biopsies [23], and bronchiolar lavage [22] sampling have been utilized to gain an overall picture of the corresponding viral reservoir. A dataset that can combine deep sequencing from multiple cellular and anatomical reservoirs will be an invaluable tool with respect to defining the exact nature of the longitudinal genetic change or variation observed in the peripheral blood and the critical cellular reservoirs during the course of HIV-1 disease.

The variable rate of sequence evolution between the differing regions of the HIV-1 genome is something that has been explored previously [31, 32]. This study confirms that *tat* exon 1 has a higher observed mutation rate (0.636 nt/kb/yr) when compared to the LTR (0.527 nt/kb/yr). The LTR and *tat* differ in that tat is expressed as a protein where the LTR is not. Because of this Tat is processed as an antigen which is recognized by the immune system. This leads to an additional selective pressure on Tat with respect to immune evasion [43] that does not exist for the LTR. Future studies will examine other HIV-1 genes in this patient population. We hypothesize that changes in mutation rate with the introduction of therapy will be more pronounced in genes encoding proteins that are directly targeted, such as the reverse transcriptase, protease, and integrase.

The mutation rates calculated above conform to previous estimates of HIV-1 mutation rates as observed by Josefsson and colleagues [36]. They found that the mutation rate of HIV-1 in untreated individuals to be 5–7 nt/kb/yr, which compares favorably to our estimate of 4–6 nt/kb/yr. However, the estimates of mutation rates in patients well-suppressed on combination ART are not directly comparable between this study and their study. Joseffson and colleagues examined the gag-pol region of the genome from the memory T-cell compartment. As such, they estimated a mutation rate of 0.01–0.15 nt/kb/yr, 50 times lower than the 3.5–4.6 nt/kb/yr that our study estimates. Their study provides an estimate of the mutation rate in the latent/persistent resting memory CD4+ T-cell population as opposed to the total cellular population in the PBMC compartment of well-suppressed HIV-1-infected patients examined in our investigations; both of which are important cellular populations to understand within the context of HIV-1 disease. Furthermore, their study specifically targeted the polymerase gene and the selective pressure imposed by ART confounds direct comparison between the two studies with respect to comparing mutation rates.

Attempts to account for these data using other clinical variables (including CD4^+^/CD8^+^ cell counts, current and peak viral loads, and age) did not have a significant impact on the result. The viral load was weakly correlated with the mutation rate but only when using a threshold at the level of detection and combined with ART status. The PreART Group was examined with respect to direct correlations between viral load and mutation rate but with no success. This could be due to numerous factors that include the fact that (1) increased viral loads may occur from a clonal expansion of a small number of infected cells leading to the development of a population of identical viral genomes leading to less overall variation [44, 45]; (2) the spike in viral load may be due to replication- or entry-incompetent virions [46]; (3) there was insufficient resolution at current time-points to measure the branch that spawned the spike in viral load; and (4) there was a general lack of statistical power when examining this relatively small number of patients. Future studies focusing on PreART time points may find additional quantitative correlations with respect to mutation rates in selected regions of the HIV-1 genome.

After initiation of ART, the mutation rate converges to an apparently patient-specific rate that stays constant over numerous years, after therapy has reduced viral load to undetectable levels. This rate was estimated to be 3.69 +/- 2.88 mutations/kb/year. It is currently unclear what parameters affect this measure, but it is likely that a combination of the specific ART regimen of each patient, patient adherence to therapy, and viral factors such as the initial viral set point all play a role. In this dataset there is no correlation between the baseline rate of genetic change and typical clinical markers such as Nadir CD4, peak viral load, or years on therapy (p>0.1). It was not possible to test the effect of specific ART regimens due to the breadth of the therapeutic approaches used in this relatively small cohort. As such, a larger dataset will be required to associate the patient-specific baseline rate of genetic change with clinical parameters such as therapeutic protocol, viral set point, as well as others.

This study is limited by the assumptions inherent in both the phylogenetic framework as well as the linear modeling. The BEAST analysis assumes that the tree is rooted at a specific time and that all observed variation results from mutations to that common founder virus. While this is likely to be true in most cases [10, 11], individuals in this cohort may have been infected numerous times. It is currently unclear how to find these instances beyond the trivial cases of superinfection with multiple HIV-1 subtypes. Additional effort needs to be expended to answer these questions to increase the likelihood of resolving these issues.

Assumptions surrounding the linear modeling may also lead to potential misinterpretations of the results. Both ART suppression and increased length of infection are correlated with a decreased mutation rate. These results may lead one to hypothesize that after enough time on therapy the mutation rate would eventually reach zero. This mirrors previous hypotheses that sufficient therapeutic control would eventually lead to viral clearance [47, 48]. However, this is not what was observed; instead the rate of genetic change appears to plateau to a patient-specific baseline mutation rate. Instead, the results presented showed that reservoirs may persist despite the most effective therapeutic control available. The results shown also confirm that the viral quasispecies in the PBMC compartment can continue to change and/or mutate with only an average of a 20% reduction of the PreART mutation rate.

With the improvement of therapeutic interventions, HIV-1 patients are living longer. As such it is important to understand the factors that affect the viral mutation rate including (1) relative frequencies abundances of quasispecies, (2) reemergence of historical sequences, and (3) the nature of the reservoirs that harbor defective and fully functional proviruses as this will be critical to treating patients in the long-term. The results reported herein indicate that even under complete therapeutic elimination of detectable viral replication, mutations within the integrated proviral DNA still occur, albeit at a lower rate. It is likely that this is a combination of active replication in areas of poor therapeutic penetrance, such as the brain, gut, or other tissues, and undetectable levels of replication in the PBMC compartment. Deep sequencing experiments on longitudinally collected samples will allow researchers to sample all quasispecies at each time-point along with relative frequencies. When combined with rich clinical information, the factors influencing each mode of variation will be examined in even greater detail.

## Ethics statement

The Drexel University College of Medicine Institutional Review Board (IRB) has approved this work under protocol 1201000748 (Brian Wigdahl, PI). All patient samples were collected under the auspices of protocol 1201000748 through written consent.
